# The First Report of Immunoglobulin G, M, and A Concentrations in Serum of European Bison and Their Changes with Age

**DOI:** 10.1155/2020/2614317

**Published:** 2020-01-23

**Authors:** Małgorzata Pomorska-Mól, Michał K. Krzysiak, Magdalena Larska, Jan Włodarek

**Affiliations:** ^1^Department of Preclinical Sciences and Infectious Diseases, Faculty of Veterinary Medicine and Animals Science, Poznań University of Life Sciences, ul. Wołyńska 35, 60-637 Poznań, Poland; ^2^Białowieża National Park, Park Pałacowy 11, 17-230 Białowieża, Poland; ^3^Institute of Forest Sciences, Faculty of Civil Engineering and Environmental Sciences, Bialystok University of Technology, Poland; ^4^Department of Virology, National Veterinary Research Institute, Al. Partyzantów 57, 24-100 Puławy, Poland

## Abstract

The age-specific reference values for immunoglobulin (Ig) serum concentrations in European bison (*Bison bonasus*) are lacking. Identification of immune alterations that accompany normal physiological aging will help assist in development of better monitoring health programs. In the present study, the age-associated changes in concentration of IgG, IgM, and IgA in serum of apparently healthy European bison of various ages were studied. The quantities of IgA, IgM, and IgG were measured by the use of a commercial ELISA kit. The serum samples originating from apparently healthy European bison (*n* = 206) were divided into the following age categories: (1) <1 year of age; (2) animals between 1 and 3 years of age; and (3) animals which have reached sexual maturity: (3a) animals between 4 and 8 years of age, (3b) animals between 9 and 15 years of age, and (3c) animals > 15 years of age. IgG was found to be predominant Ig in the serum regardless of the age of the animals. The significant positive correlation between IgG absolute and relative concentration and the age of animals was found. The absolute concentration of IgM did not differ significantly during the lifespan; however, the negative correlation was observed between percentage of IgM and European bison's age. IgA represented the least class of serum Ig. Total serum concentration of analyzed Ig also significantly increased with age. No gender-related differences were detected. Our findings represent a meaningful contribution to the studies on the immunity of European bison and effect of age on the immunoglobulin level. Our results would be useful for veterinarians and researchers in the studies with this animal's species.

## 1. Introduction

Development of the immune system is not complete at the time of birth in humans and various animal species [1–3]. The maturation and further development of the immune system occur during the first weeks of life. Therefore, the values of immune parameters vary during neonatal and later periods of life, which is observed in many animals' species [1, 4, 5], as well as in humans [6, 7]. Furthermore, the age-related changes in humoral and cell-mediated immunity have been suggested to contribute to the decrease in immune competency in aged individuals [8–10]. Differences observed in peripheral blood lymphocyte subpopulations and serum concentrations of immunoglobulins in children compared to adults have prompted similar studies in animals [1–3, 11–15]. The relative values of *γ*-globulins were significantly lower in younger calves and dogs compared to the older ones [9, 10]. In contrast, in horses, serum concentration of IgG, IgM, or IgA did not differ with age [8].

Recent studies indicate that every component of the immune system undergoes age-associated changes, suggesting that the immune aging is a result of the continuous adaptation of the organism to the environment as well as overall and over time deterioration [2, 12, 15, 16].

The age-specific reference values for immunoglobulin (Ig) serum concentrations in European bison (*Bison bonasus*) are lacking but are essential for more deep studies on their health status and immunity. The immunological data relating to this species could form the basis for the further studies on physiology and pathology. Identification of immune alterations that accompany normal physiological aging in European bison will help assist in development of more appropriate monitoring health programs.

Therefore, the present study was designed to characterize age-associated changes in absolute and relative concentration of IgG, IgM, and IgA, representing the humoral component of acquired immunity, in serum of apparently healthy European bison (*Bison bonasus*) of various ages. To our knowledge, this is the first report of the concentration and percentage of IgG, IgM, and IgA in serum of European bison, as well as age-related changes observed in apparently healthy animals during the lifespan.

## 2. Material and Methods

### 2.1. Samples

A total of 206 serum samples were obtained from European bison (*n* = 206) between 2014 and 2017. The particular locations of animals were as follows: Białowieża Forest (*n* = 85, free ranging), Pszczyna (*n* = 46, captive), Bieszczady Mountains (*n* = 11, free ranging), Niepołomice (*n* = 26, captive), Smardzewice (*n* = 17, captive), Borecka Forest (*n* = 11, free ranging), Gołuchów (*n* = 4, captive), Bałtów (*n* = 3, captive), Knyszyńska Forest (*n* = 2, free ranging), and Wałcz (*n* = 1, captive) which represent the main populations of European bison in Poland. Slightly more female European bison (*n* = 115) were sampled than males (*n* = 91). The age of the animals ranged between 2 months and 21 years. The animals were divided into three age categories according to the generally accepted division by Krasińska and Krasiński [17] as follows: (1) calves < 1 year of age (*n* = 22), (2) young animals between 1 and 3 years of age (*n* = 74), and (3) adult animals which reached sexual maturity further divided into three age subcategories as follows: (3a) adult animals between 4 and 8 years of age (*n* = 59), (3b) adult animals between 9 and 15 years of age (*n* = 31), and (3c) old animals > 15 years of age (*n* = 20). The detailed characteristic of groups used in the study is presented in Table 1.

Blood samples originated from apparently healthy European bison, which were pharmacologically immobilized for transportation, collaring, or diagnostic purposes according to the previously described protocols [18]. The animals were sampled in accordance with the appropriate regulations and permits (*Polish General Directorate for Environmental Protection: Regulations DOP-OZGIZ.6401.06.7.2012.1* s *and DOPOZ. 6401.06.7.2012.1 s1.Warsaw, 2012*; *Polish General Directorate for Environmental Protection: Regulations DZP-WG.6401.06.23.2014.km2.*; and *Polish Ministry of the Environment, Regulation: DLP-III-0771-5/42173/14/ZK*).

The blood from the jugular vein was collected into sterile tubes with clot activator for serum separation. The poor quality samples were excluded prior to the study for ensuring the high reliability of results. Serum was harvested from the blood samples by centrifugation (3000 × *g*, 15 min, room temperature) and stored at -20°C for further analysis (not longer than one month).

### 2.2. Determination of Immunoglobulin Concentration

The quantities of IgA, IgM, and IgG in serum were measured by the use of a commercial ELISA kit dedicated to cattle (ELISA Quantitation Kit, Bethyl Laboratories Inc., USA). Prior to analysis, all samples were diluted in Tris-buffered saline (TBS) with 0.05% Tween 20. Depending on the expected analyte concentration, the dilution was as follows: 1 : 5000 for IgA and IgM and 1 : 100 000 for IgG. The sheep antibovine IgA, IgM, or IgG-Fc fragments were diluted in 0.05 M sodium carbonate solution (pH 9.6) to the final concentration of 1%, and the plates were coated with 100 *μ*L of the above solutions for 60 minutes at 20°C. Thereafter, the plates were blocked for 30 minutes at 20°C with TBS with 0.05% Tween 20. Diluted samples were added in duplicates to the plates (100 *μ*L·well^–1^) and incubated for 1 h at 20°C. The plates were subsequently incubated for 1 h at 20°C with peroxidase-labeled sheep antibovine IgA, IgM, or IgG-Fc fragment and for 15-20 minutes in the dark at 20°C with 100 *μ*L·well^–1^ of substrate (3,3′,5,5′-tetramethylbenzidine). Between each step, plates were washed three times with TBS containing 0.05% Tween 20, with the use of a microplate washer (Autura 1000, Mikura Ltd., UK). The colored reaction was stopped with 100 *μ*L·well^–1^ of 2 M H_2_SO_4_ solution, and the absorbance was recorded at 450 nm using an ELISA plate reader (Multiskan RC, Labsystems, Finland).

In each analysis, serial dilutions of standard samples of bovine reference serum were tested in order to obtain a calibration curve, which was then computer-adjusted (with the use of the FindGraph software). The values of Ig concentration in the tested samples were calculated from the calibration curve by the same software. Ranges of detection of ELISA kits were as follows: 15.62–1000 ng/mL for IgA and IgM and 7.8–500 ng/mL for IgG. The results obtained (in ng/mL) were multiplied by the appropriate dilution factor and expressed as mg/mL.

### 2.3. Statistical Analysis

Data were subjected to Shapiro-Wilk's test (*p* < 0.05) of normality and Levene's test (*p* > 0.05) of equal variances. For nonnormal distribution of data, the nonparametric Kruskal-Wallis test with post hoc multiple comparisons for comparison of all pairs (mean Ig concentration in various groups) was used for statistical analysis of differences in serum immunoglobulin concentration between age and sex. The Spearman rank correlation test was used to calculate the correlation between immunoglobulin concentration and age of animals. Data were categorized to age and sex. For all analyses, *p* < 0.05 was considered significant. Statistica 8.0 (StatSoft, Poland) was used to perform all analyses.

## 3. Results

**Figure 1:**
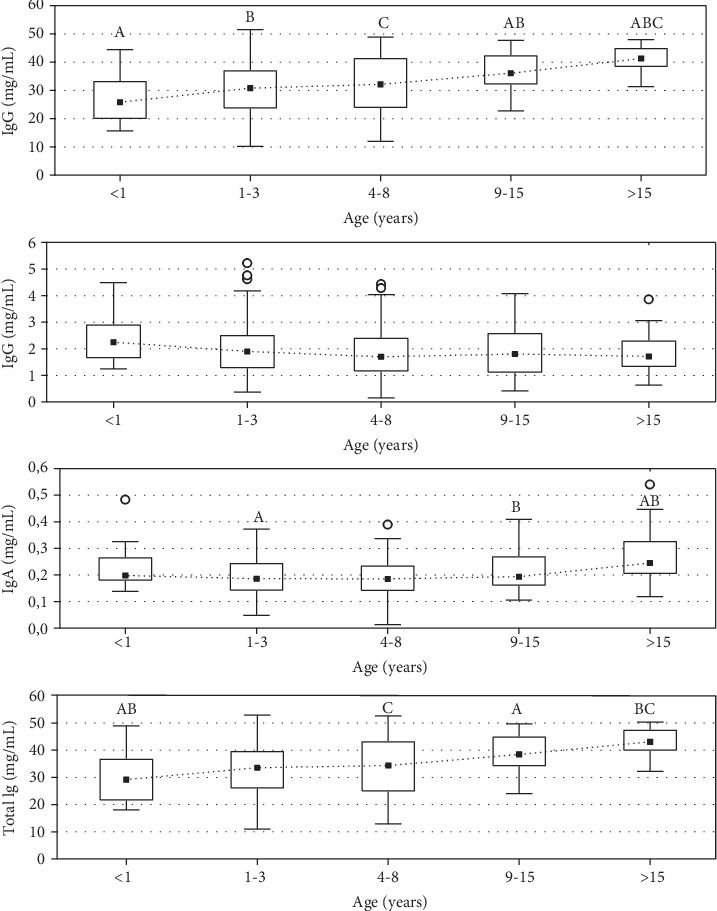
Distribution of the absolute concentration (mg/mL) of IgG, IgM, and IgA and total concentration of analyzed Ig (total Ig) in serum of apparently healthy European bison of various ages. Dotted line represents median values, boxes represent the 25th and 75th percentiles, and error bars indicate the 5th and 95th percentiles for each immunoglobulin class. The circles represent outliers. ^abc^Values with the same superscript differ significantly between age groups within the same class of immunoglobulin (*p* < 0.05).

**Figure 2:**
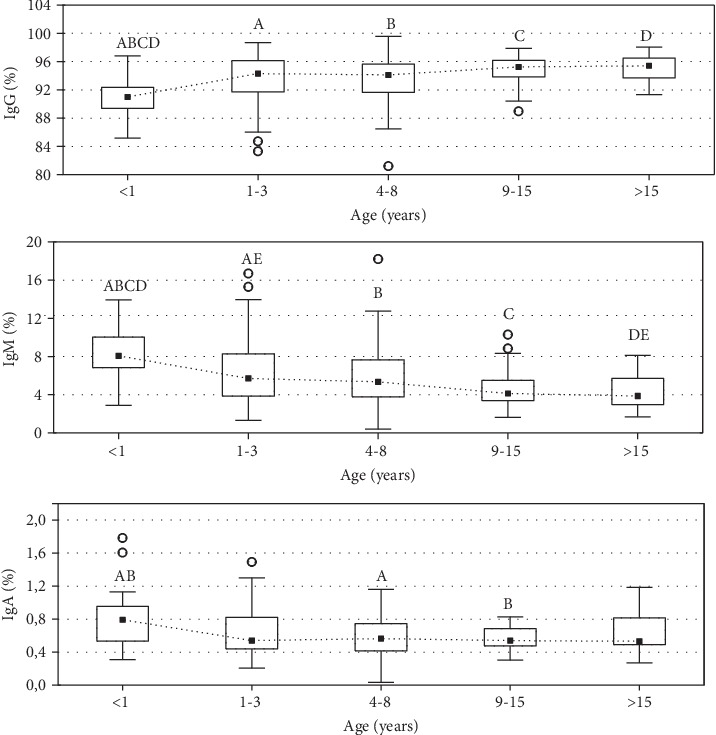
Distribution of the relative concentration (%) of IgG, IgM, and IgA in serum of apparently healthy European bison of various ages. Dotted line represents median values, boxes represent the 25th and 75th percentiles, and error bars indicate the 5th and 95th percentiles for each immunoglobulin class. The circles represent outliers. ^abcde^Values with the same superscript differ significantly between age groups within the same class of immunoglobulin (*p* < 0.05).

In our study, several age-related changes in investigated immune parameters were observed. The data referring to the mean (±SD) absolute (mg/mL) and relative (%) concentration of IgG, IgM, and IgA and total concentration of analyzed Ig (mg/mL) in serum of apparently healthy European bison of various ages are presented in Tables 2 and 3. The ranges of serum concentrations of IgG, IgM, and IgA and total concentration of analyzed Ig observed throughout the lifespan of animals are presented in Figures 1 and 2.

**Figure 3:**
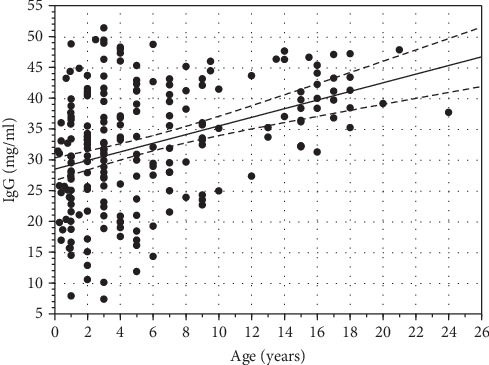
Relationship between absolute concentration of IgG in serum and age of European bison. Dotted lines represent 95% confidence intervals (*R*_Spearman_ = 0.376, *p* = 0.0005).

IgG was found to be predominant immunoglobulin in the serum regardless of the age of the animals (it constituted from 81.12% to 98.4% of total concentration of analyzed Ig depending on the animal). IgG absolute concentration increased with the age of animals (Figure 1). The relative concentration was also significantly lower in the youngest animals compared to older ones (Figure 2). The significant positive correlation between IgG absolute and relative concentration in serum and the age of animals was statistically proven (*ρ*_Spearman_ = 0.376, *p* = 0.0005 and 0.289, *p* = 0.0002, respectively) (Figure 3). The variability of IgG levels was the highest in the animals between 1 and 3 years old, while the lower variability was observed in animals older than 15 years.

**Figure 4:**
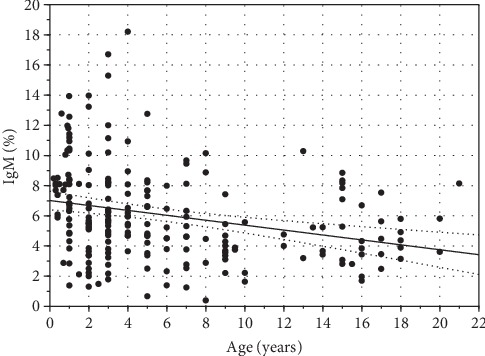
Relationship between relative concentration of IgM in serum and age of European bison. Dotted lines represent 95% confidence intervals (*R*_Spearman_ = 0.361, *p* < 0.0001).

The absolute concentration of IgM in serum of clinically healthy European bison remained relatively stable and did not differ significantly during the lifespan (*p* ≥ 0.05) (Figure 1). In contrast, the significantly lower frequency of IgM was observed in adult animals compared to younger ones (*p* < 0.05) (Figure 2), and negative correlation was observed between the percentage of IgM and European bison's age (*R*_Spearman_ = −0.346, *p* < 0.0001) (Figure 4). The maximum variation in concentration of IgM was observed between the second and eighth years of life, while in the oldest animals, the variation was the lowest.

IgA represented the least class of serum immunoglobulins in European bison, with the frequency between 0.05% and 1.49%. There was no clear trend observed in both absolute and relative number of IgA during the lifetime. The significant differences between particular age groups are indicated in Figure 2. Similarly as for IgM, the variability of IgA levels was the highest in the animals between 1 and 8 years old.

Total concentration of analyzed serum Ig, expressed as the sum of IgG, IgM, and IgA concentrations calculated for each animal, significantly increased with age (*p* < 0.05). The mean total concentration of analyzed Ig in animals older than 15 years was almost 1.5 times higher than that in the youngest European bison (<1 year of age), and the little variation within the age category has been found. The significant positive correlation between total concentration of analyzed Ig in serum and the age of animals was noted (*R*_Spearman_ = 0.361, *p* < 0.0001). This increase is mainly due to the increase in IgG concentration in aged bison, since the most significant raise within age was found in concentration of IgG.

No gender-related differences were found within all age categories (*p* ≥ 0.05) regarding all Ig studied, including total concentration of analyzed Ig.

## 4. Discussion

In the present study, the age-specific values of IgG, IgM, and IgA serum concentrations in the apparently healthy European bison were investigated using bovine commercial ELISA assays. Since the European bison is closely related to cattle (*Bos taurus*), the bovine-specific diagnostic assays have been successfully used previously for testing samples collected from European bison [19–21].

According to the authors' knowledge, no research to characterize dynamic changes of Ig concentrations in serum has been undertaken in European bison to date. Identification of normal changes of the immune system in young or geriatric European bison is a prerequisite for development of medical intervention protocols for diseases or disorders characterized by physiologic and pathologic age-associated immune alterations.

Our data yield clinically applicable findings for the serum Ig concentration in different age groups of European bison and demonstrate that the absolute and relative concentration of serum IgG and total concentration of analyzed Ig are significantly influenced by age (*p* < 0.05). Moreover, the significant age-related trend regarding frequency of IgM was observed. This shift in the serum IgG concentration suggests that the age of the animals should be taken into consideration when interpreting serum IgG concentration. The increase of IgG concentration and frequency with age may reflect the accumulation of chronic inflammatory conditions with aging or it may be the result of repeated contact with pathogens, resulting in a strong secondary immune response. The lack of such association for IgM and IgA absolute level may be associated with a significantly shorter serum half-life. In humans, similarly as in our study, IgG absolute concentration also increased with age, whereas IgM absolute concentration remains unchanged [22]. Changes in serum immunoglobulin concentrations in humans have been also reported by Buckley & Dorsey [23]. They found significant age-related differences between all Ig classes. However, the maximum serum IgG concentrations were reached in the third decade in humans followed by a significant decrease from the third through the sixth decades. Similarly as in the present study, in humans, age-related changes in serum IgA were not significant. In mice, various isotype immunoglobulin absolute levels also were shown to increase with age [24]. The higher IgG concentration (absolute and relative) has been also observed in older pigs compared to younger pigs and in cattle [2, 9]. In addition, the significant increase in *γ*-globulin concentration in the older dogs has been reported previously [10]. Similar findings were reported by Weaver et al. [25], who stated that in the serum of calves, there is very low concentration of *γ*-globulins and that its concentration increased with age. Chaudhary et al. [26] found that the concentration of total proteins, albumin, and *γ*-globulins was higher in the adult camels than in camel calves. It has been suggested that the lower concentration of immunoglobulins in camel calves is caused by the immaturity of the lymphoid system [26]. In contrast, Flaminio et al. [27] reported that in goat, both serum IgG and IgM concentration increased linearly with age. When the immune system matures, the production of immunoglobulins is more efficient and higher Ig concentrations are present in the serum [26]. Data presented by McFarlane et al. [8] indicate that age-dependent changes in cell-mediated and humoral development existed also in horses. Kaneko et al. [28] also reported that over the lifespan of domestic animals, there is a general increase in globulin concentration with advancing age. Age thus is important consideration in the interpretation of results of serum immunoglobulin concentration.

Species analogies with regard to aging as presumed to exist between man, laboratory, rodent, and other animal species also seem to be applicable to European bison [1, 2, 11, 14, 15].

In the present study, in agreement with previous findings in humans, we did not show any gender-related effect on serum IgA, IgM, IgG, and total Ig levels [11].

## 5. Conclusion

On the basis of our study, we state that in the evaluation of health status and immune disorders in European bison, similarly as in other animal species, it is of great importance to interpret the results of studied immunological parameters with regard to physiological processes of development, functional immaturity of the immune system, and immunological reactions which occur in the body during normal development. Results of the present study also suggest that valid contrasts of serum immunoglobulin concentrations (absolute and relative) between healthy and diseased animals are not possible without adequate control of biologic variation related to age. Interpretations based on data in which age effects are not controlled are at risk. Because the concentration of immunoglobulins in serum has not been previously investigated in European bison, the obtained data provide original knowledge for veterinarians in the evaluation of various physiological and pathological conditions for further specific laboratory examinations. Our results would be also useful for researchers in the studies with this animal's species. Our findings represent a meaningful contribution to the studies on the immunity of European bison and effect of age on the serum relative and absolute concentration of investigated immunoglobulins.

## Figures and Tables

**Table 1:** The detailed characteristic of groups of European bison used in the study.

Group	*n*	Mean age in years (SD)	Sex Female/male
1	22	0.55 (0.29)	8/14
2	74	2.09 (0.80)	44/30
3a	59	5.47 (1.38)	31/28
3b	31	11.76 (2.59)	20/11
3c	20	17.37 (1.53)	12/8

SD: standard deviation.

**Table 2:** Mean (±) absolute concentration (mg/mL) of IgG, IgM, and IgA and total concentration of analyzed Ig (total Ig) in serum of apparently healthy European bison of various ages.

Age category (years)	*n*	Absolute concentration (mg/mL) (mean ± SD)			
		IgG	IgM	IgA	Total Ig
<1	22	27.57^a^ ± 8.54	2.48 ± 0.92	0.23 ± 0.07	30.28^ab^ ± 8.95
1-3	74	30.84^b^ ± 9.81	2.05 ± 1.09	0.19^a^ ± 0.07	32.89^c^ ± 10.07
4-8	59	32.35^c^ ± 9.94	1.92 ± 1.05	0.19 ± 0.07	34.46 ± 10.29
9-15	31	36.34^ab^ ± 7.03	1.89 ± 0.97	0.21^b^ ± 0.08	38.44^a^ ± 7.49
>15	20	41.39^abc^ ± 4.41	1.87 ± 0.84	0.27^ab^ ± 0.10	43.53^bc^ ± 4.69

^abc^Values with the same superscript differ significantly (*p* < 0.05).

**Table 3:** Mean (±SD) relative concentration (%) of IgG, IgM, and IgA (%) in serum of apparently healthy European bison of various ages.

Age category (years)	*n*	Relative concentration (%) (mean ± SD)		
		IgG	IgM	IgA
<1	22	90.75 ± 2.81^abcd^	8.42 ± 2.57^abcd^	0.82 ± 0.36^ab^
1-3	74	93.46 ± 3.41^a^	6.53 ± 3.40^ae^	0.64 ± 0.28
4-8	59	93.62 ± 3.18^b^	5.78 ± 3.06^b^	0.58 ± 0.23^a^
9-15	31	94.60 ± 2.21^c^	4.84 ± 2.13^c^	0.55 ± 0.15^b^
>15	20	95.10 ± 1.86^d^	4.26 ± 1.81^de^	0.62 ± 0.23

^abcd^Values with the same superscript differ significantly (*p* < 0.05).

## Data Availability

The data used to support the findings of this study are available from the corresponding author upon request.
